# Family Risk Factors That Jeopardize Child Development: Scoping Review

**DOI:** 10.3390/jpm12040562

**Published:** 2022-04-01

**Authors:** Aida Simões, Saudade Lopes, Maria dos Anjos Dixe, Júlio Belo Fernandes

**Affiliations:** 1 Escola Superior de Saúde Egas Moniz, 2829-511 Almada, Portugal; aidasimoes@gmail.com; 2 Centro de Investigação Interdisciplinar Egas Moniz (CiiEM), 2829-511 Almada, Portugal; 3 Center for Innovative Care and Health Technology (ciTechcare), School of Health Sciences, Polytechnic of Leiria, 2411-901 Leiria, Portugal; saudade.lopes@ipleiria.pt (S.L.); maria.dixe@ipleiria.pt (M.d.A.D.); 4 Grupo de Patologia Médica, Nutrição e Exercício Clínico (PaMNEC)—Centro de Investigação Interdisciplinar Egas Moniz (CiiEM), 2829-511 Almada, Portugal

**Keywords:** child, child abuse, maltreatment, neglect, risk factors, family, caregivers, child protective services

## Abstract

The obligation to protect children is defined by law. However, there is fragility in identifying actual or potential situations that jeopardize their development. This review aims to identify the family risk factors that jeopardize child development. A scoping review was conducted following the Joanna Briggs Institute for Evidence-Based Practice framework and the 2020 Preferred Reporting Items for Systematic Reviews and Meta-analyses (PRISMA) statement. The research was carried out on the electronic databases PubMed, CINAHL, Nursing & Allied Health Collection: Comprehensive, MEDLINE Complete, and MedicLatina, with a time limit of 2010 to 2021. The search was restricted to documents written in Portuguese, English, and French. A total of 3998 articles were initially identified. After selecting and analysing, 28 risk factors were extracted from 29 articles. Four categories of risk factors were identified—namely, patterns of social and economic interaction, family characteristics, caregiver’s characteristics, and parenting. The results of this review allow the identification of family risk factors that jeopardize child development. This is significant for Child Protective Services workers as they carry out their risk assessments. This assessment is the first step in avoiding an accumulation of harm to at-risk children and allowing the development of interventions for minimising harm’s impact on children’s development.

## 1. Introduction

The family is a micro-system with an organization, both structural and functional. Each family member plays a socially defined role, referring us to a space of affection, harmony, and protection between its members. The family environment has been the subject of several studies on its implications for children’s development and can be conceptualized as a resource or adversity [1].

Even though most children grow in desirable environments, a minority are victims of multiple types of maltreatment by their parents or caregivers [2].

When children are at risk of or are experiencing neglect or any other form of maltreatment, Child Protective Services (CPS) becomes involved in investigating the allegations. The CPS is a branch of the state’s social services department that assesses, investigates, and intervenes in cases of an allegation of child abuse and neglect. The main focus is on promoting child’s rights and safety and providing support for parents to strengthen families and promote a safe, nurturing environment for children [3]. While effective engagement is an essential component for helping children and their families, this process presents an ongoing challenge [4]. There is an underlying tension caused by the controlling function inherent to the CPS and the contribution to developing families’ skills to produce better outcomes for children [5].

Dealing with the duality of this relationship can be challenging for families and CPS, given the expectations that CPS workers will engage in conflicting roles of supporting families on the one hand and ensuring the child’s safety on the other. In addition, having the right to remove a child from their home causes a level of mistrust throughout the interaction [6].

For CPS to investigate any allegations of children who are at risk of or who are experiencing any type of abuse or neglect, the initial assessment is crucial. This assessment is performed by CPS workers and enables the collection of information that confirms or disproves the veracity of the allegations [7,8].

The CPS workers come from various professional areas, and in practice, it is verified that their assessments are based on criteria arising from their fields of training and expertise. This may lead to difficulty in gathering information, and consequently, it may hinder decision-making. 

Previous research has revealed several risk factors frequently related to child maltreatment. For example, a study assessing the risk of child abuse showed a higher risk for mothers with lower education and social support [9]. Another study reviewed CPS reports from 2006 to 2008 for families in Connecticut’s child abuse prevention program and identified several family risk factors—namely, histories of CPS, domestic violence, mental health, sexual abuse, substance abuse, and criminal involvement [10]. Children in families and environments exposed to these factors have an increased probability of experiencing neglect or any other type of abuse [11,12]. Although several studies approach the risk factors for child maltreatment and the improvements in risk assessment, recent research has not adequately looked at the factors related to the family. 

A better understanding of family risk factors can support CPS workers in the identification of stressors and situations that put children at risk of neglect or abuse, allowing CPS workers to intervene before maltreatment occurs. Considering this gap in evidence, this review aims to identify the family risk factors that jeopardize child development.

## 2. Methods

This scoping review report was drawn based on the 2020 Preferred Reporting Items for Systematic Reviews and Meta-analyses (PRISMA) statement [13]. In addition, our methods followed the Joanna Briggs Institute for Evidence-Based Practice framework [14]. According to PCC, the following research question was defined: What are the family risk factors that jeopardize child development?

The PubMed, CINAHL, Nursing & Allied Health Collection: Comprehensive, MEDLINE Complete, and MedicLatina databases were searched using a combination of concepts in line with the DeCS/MeSH terms. The search equation was: ((Social service Or Social work OR Protection service) AND (Family OR Caregiver) AND (Neglect OR Abuse OR Maltreatment) AND (Child OR Child, preschool OR Adolescent)).

The selection criteria were: documents written in Portuguese, English, and French, published between 2010 and 2021, which addressed or referred to the family risk factors that jeopardize child development. All documents that did not meet the selection criteria were excluded from the review.

Two researchers independently carried out the search, selection, and extraction of data to increase consistency.

The article selection was performed in three phases. In the first phase, the title was analysed, followed by the abstract analysis, and, finally, the selected articles were read in full. When it was unclear whether an article fit this review, it went to the following analysis stage. Disagreements between reviewers were resolved through discussion. In occasional situations, if any doubt or inconsistency was present, the issues were discussed by all researchers.

Data were extracted using an instrument designed for data extraction, considering the defined research questions. Finally, all authors discussed the final extraction chart.

A data-driven thematic analysis adopted from Braun, Clarke, Hayfield, and Terry’s guidelines [15] was undertaken. Two researchers reviewed data independently and manually coded using inductive analysis to identify common themes across the collected data. Data were separated into meaningful units of words or phrases on the same topic. Then, codes were assigned to the meaningful units, and categories were identified, reflecting differences and similarities in data. The two researchers compared their findings to finalize this analysis process, and discussion resolved any discrepancies. Finally, the whole research team reviewed the final findings.

From the analysis, the family risk factors were grouped under different categories.

## 3. Results

**Figure 1:**
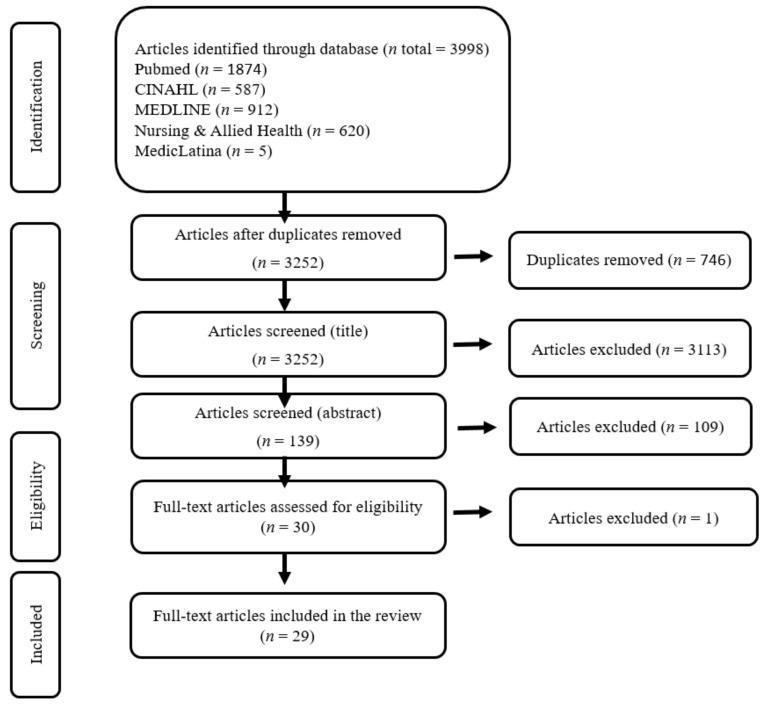
PRISMA flow chart.

The study selection process is summarized below in the PRISMA flow chart (Figure 1). Initially, 3998 documents were identified in the different databases. Then, after analysing the complete texts, 29 articles (Table 1) presented knowledge that allowed answering the research question.

A total of twenty-nine studies published between 2010 and 2021 were selected for final analysis. Regarding the country where studies were applied, we verified that the majority of studies were from the USA [4,8,10,11,12,16,17,19,20,21,22,26,29,34,35,38], eight were from Europe [9,23,24,25,28,30,31,33], two were from Canada [27,37], one was from China [18], one was from Australia [32], and another was from Iran [36].

The content analysis of the data in the articles allowed the identification of twenty-eight family risk factors that jeopardize child development, which were grouped into four categories. These results are presented in Table 2.

## 4. Discussion

Experiencing physical and emotional maltreatment in childhood can lead to the development of a broad range of long-term health conditions that can manifest as educational difficulties, anxiety, depression, trouble forming and maintaining relationships, drug use, and suicide attempts [39,40,41,42]. Therefore, it is essential to identify and, if possible, eliminate or mitigate factors that jeopardize child development.

This review helps to understand the family risk factors that jeopardize child development. Research in this area is crucial, especially as new data reveal the true impact of the cumulative harm of repeated abusive and neglect parenting practices on early brain development, emotional regulation, and cognitive and social development. The results from this review support prior research findings that when assessing families with chronic neglect and abusive practices, CPS deals with significant and multiple risk factors [43]. 

Multiple risk factors emerged from the review. The first category of risk factors demonstrates the relationship of social and economic interaction patterns in child development. The risk factors that encompass this category are hazardous housing, low social support, no means of transportation, mismanaged finances, and the presence of violence in the community and neighbourhood.

Problems in accessing basic needs, including income, employment, adequate housing, childcare, and transport, are risk factors. In addition, mismanaged finances are considered a risk factor that contributes to high family stress levels. In combination with other factors, low economic resources contribute to an increased propensity for child abuse and neglect [4,8,9,28,36,38].

The existence of means of transportation is significant for parents to have access to medical appointments, school activities, job interviews, and transport of essential goods for household activities [21].

Concerning family social exclusion, Macdonald et al. [24] concluded that, by itself, it is a risk factor for the situations of neglect and abusive practices by families.

We found that specific family characteristics such as a history of allegations, domestic violence, family disagreements and conflicts, single-parent families, multiple caregivers, the existence of stepmothers and stepfathers, multi-child families, being the firstborn, and poor communication between separated parents might also be risk factors during child development.

Previous studies found that family characteristics such as having a history of allegations, including a history of abuse and neglect, are risk factors for the child’s development [10,21,22,25,26]. Furthermore, families with multiple allegations, either for different children or referring the same child several times, are also associated with child abuse and neglect [26]. In addition, children from single-parent families are more likely to be neglected than children from two-parent families [10,11,18,20,22,25,28]. Thus, there is broad agreement that child maltreatment should be analysed as a problem in the family system, although research focuses on the mother or father [44].

Ineffective communication is a barrier between the child’s caregivers, especially when the parents are separated, with difficulties understanding care [21,27,36]. When the most consistent pattern in the parents’ relationship involves violence, children exposed to it are more likely to abuse their partner physically. There is a risk of intergenerational transmission of partner violence, which increases when either parents or caregivers are violent [44].

The category caregiver’s characteristics encompasses risk factors that include history of inflicting maltreatment, having been neglected, criminal record, alcohol and/or drug abuse, mental health problems, low level of education, lack of cooperation with CPS, being under 18 years old, and unintended pregnancy.

Several authors frame the use of alcohol or drugs in changes in family functioning [4,8,10,11,22,24,25,26,28,29,32,33,36]. These data are corroborated by a study by Felitti et al. [45], which assessed the adverse childhood experiences of 13,494 individuals. These researchers identified psychological, physical or sexual abuse, domestic violence, and living with substance-abusing and mentally ill family members as risk factors for the child’s development.

Previous studies identified that physical illness and physical, intellectual, or cognitive impairments might be considered risk indicators. Laslett, Room, and Dietze [46] conclude that mental or physical illness and alcohol or drug abuse are caregiver characteristics that should be considered risk factors for the child’s development.

The caregivers’ low level of education is a predictor of abuse and neglect [9,10,11,22,23,25,26]. A study conducted by Hirsch et al. [26] found that a low level of education was a constant, with 79% of participants having no more than primary education, regardless of whether the associated issue was abuse, neglect, or other forms of maltreatment.

Another risk factor identified in this review was the caregiver’s lack of cooperation with Child Protective Services. This lack of collaboration jeopardizes the child’s well-being and increases the risk for abuse and neglect [9,12,23].

The last category shows the risk factors associated with parenting that may jeopardize the child’s development—namely, not meeting the child’s unique needs, unrealistic expectations towards the child, or negative attitudes towards the child, abusive and mistreating practices, multiple trips to children’s emergency room, and parents absent in childrearing.

Negative attitudes towards the child are considered to be psychologically toxic parenting behaviours [27]. Along the same line, Zimmermann et al. [28] identified that one of the risk factors for abuse or neglect was perceiving the child as a burden.

There is a significant reduction in the parental skills and commitment of the caregiver’s motivation for parenting when physical abuse and child neglect are involved, either due to lack of supervision, unmet basic needs, unsafe home environment, and lack of health care [4]. In addition, emotional sensitivity, parental guidance, the quality of the affective relationship, and the failure to promote the children’s autonomy severely compromise cognitive development and reduce the affective sphere, translating into changes in the child’s behaviour [28].

CPS assessment needs refinement to better identify cases of child abuse and neglect. This process can be accomplished by introducing an evaluation of family risk factors specific to child neglect and abusive practices [34]. Furthermore, the assessment should be holistic—not focusing on a single factor but considering the accumulation of harm perceived through the various interactions with CPS [43]. 

Current CPS practices focused on determining the risk of harm associated with the present allegations, not considering past patterns of abuse or neglect. A shift in thinking is necessary to minimize the harmful impact of recurrent neglect over time. CPS can achieve better results by adding a family risk factors assessment in its evaluation processes.

As critics have argued, the introduction of screening tools in CPS assessment processes needs to be followed by training and buy-in from frontline staff [47]. Therefore, training regarding family risk factors and their implications on the child’s development should be mandatory for all CPS professionals who handle the allegations.

The results from this review can be helpful for CPS workers when assessing children and families to identify stressors that place a child at risk for child maltreatment. A comprehensive family assessment is essential for identifying children who have experienced or who are at risk of maltreatment. This assessment enables CPS workers to identify and prevent further abuse or neglect and support and improve parental abilities to guarantee a safe and nurturing environment that allows the child’s development.

Our results can also be significant for future research, as they can be used as a foundation on which to develop screening tools for assessing family risk factors that jeopardize child development.

This research has several limitations. First, the researchers’ choice to develop a scoping review combined with a data-driven thematic analysis instead of a meta-analysis may impact data reliability. Second, the databases’ restrictions and imposed time limits may influence the results obtained. Third, we have to consider the exclusion of studies written in languages other than English, Spanish, or Portuguese. These methodological choices may have led to some relevant studies being excluded.

## 5. Conclusions

The result of this research intends to contribute to allow CPS to adequately assess the presence of risk factors that jeopardize child development. This initial assessment is a fundamental step that enables CPS to collect information that confirms or disproves an allegation’s veracity and then act to minimize the impact of the risk factor on the child’s development. This review addresses a critical evidence gap regarding the family risk factors that jeopardize child development. It allowed the identification of several articles from which family risk factors were extracted, falling into four categories: social and economic interaction patterns, family characteristics, caregiver’s characteristics, and parenting. The identification of these four categories that comprise twenty-eight family risk factors can help CPS workers identify stressors and situations that put children at increased risk for maltreatment. However, despite the usefulness of these results, we must emphasize that although certain factors might be present in families where neglect and abuse occur, it should not be assumed that the presence of these factors causes child maltreatment.

## Figures and Tables

**Table 1:** Selected articles.

Study (S)/Authors/Year	Title
S1—Donohue et al. (2016) [16]	Development and initial psychometric examination of the Home Safety and Beautification Assessment in mothers referred to treatment by child welfare agents.
S2—Gurwitch et al. (2016) [17]	Child-Adult Relationship Enhancement (CARE): An evidence-informed program for children with a history of trauma and other behavioural challenges.
S3—Casillas, Fauchier, Derkash, and Garrido (2016) [11]	Implementation of evidence-based home visiting programs aimed at reducing child maltreatment: A meta-analytic review.
S4—Peng et al. (2015) [18]	A systems approach to addressing child maltreatment in China: China needs a formalised child protection system.
S5—Loman and Siegel (2015) [4]	Effects of approach and services under differential response on long-term child safety and welfare.
S6—Winokur, Ellis, Drury, and Rogers (2015) [19]	Answering the big questions about differential response in Colorado: safety and cost outcomes from a randomised controlled trial.
S7—Jones (2015) [20]	Implementation of differential response: a racial equity analysis.
S8—Fuller, Paceley, and Schreiber (2015) [21]	Differential Response family assessments: listening to what parents say about service helpfulness.
S9—Duffy, Hughes, Asnes, and Leventhal (2015) [10]	Child maltreatment and risk patterns among participants in a child abuse prevention program.
S10—Goltz, Mena, and Swank (2014) [22]	Using growth curve analysis to examine challenges in instrumentation in longitudinal measurement in home visiting.
S11—Schneiderman, Hurlburt, Leslie, Zhang, and Horwitz (2012) [12]	Child, caregiver, and family characteristics associated with emergency department use by children who remain at home after a child protective services investigation.
S12—Benbenishty et al. (2015) [23]	Decision making in child protection: An international comparative study on maltreatment substantiation, risk assessment and interventions recommendations, and the role of professionals’ child welfare attitudes.
S13—Macdonald et al. (2014) [24]	THE SAAF STUDY: evaluation of the Safeguarding Children Assessment and Analysis Framework (SAAF), compared with management as usual, for improving outcomes for children and young people who have experienced, or are at risk of, maltreatment.
S14—Glad, Jergeby, Gustafsson, and Sonnander (2014) [25]	Social worker and teacher apprehension of children’s stimulation and support in the home environment and caregiver perception of the HOME Inventory in Sweden.
S15—Hirsch, Yang, Font, and Slack (2015) [26]	Physically hazardous housing and risk for child protective services involvement.
S16—Malo, Moreau, Lavergne, and Hélie (2016) [27]	Psychological maltreatment, the under-recognised violence against children: a new portrait from Quebec.
S17—Zimmermann et al. (2016) [28]	Growing up under adversity in Germany: Design and methods of a developmental study on risk and protective mechanisms in families with diverse psychosocial risk.
S18—Ben-David (2016) [29]	A Focus on Neglect: Comparing the Characteristics of Children and Parents in Cases of Neglect, Abuse, and Non-CAN (Child Abuse and Neglect) in Israeli Rulings on Termination of Parental Rights.
S19—Liel et al. (2020) [30]	Risk factors for child abuse, neglect and exposure to intimate partner violence in early childhood: Findings in a representative cross-sectional sample in Germany.
S20—Ajduković, Rajter, and Rezo (2018) [9]	Individual and contextual factors for the child abuse potential of Croatian mothers: The role of social support in times of economic hardship.
S21—Vial et al. (2020) [31]	Exploring the interrelatedness of risk factors for child maltreatment: A network approach.
S22—Laslett, Room, Dietze, and Ferris (2012) [32]	Alcohol’s involvement in recurrent child abuse and neglect cases.
S23—Vincent and Petch (2017) [33]	Understanding child, family, environmental and agency risk factors: findings from an analysis of significant case reviews in Scotland.
S24—Logan-Greene and Jones (2018) [34]	Predicting chronic neglect: Understanding risk and protective factors for CPS-involved families.
S25—Gifford, Eldred, Sloan, and Evans, (2016) [35]	Parental Criminal Justice Involvement and Children’s Involvement With Child Protective Services: Do Adult Drug Treatment Courts Prevent Child Maltreatment?
S26—Nilchian et al. (2012) [36]	Evaluation of factors influencing child abuse leading to oro-facial lesions in Isfahan, Iran: A qualitative approach.
S27—Palusci and Ilardi (2020) [8]	Risk Factors and Services to Reduce Child Sexual Abuse Recurrence.
S28—McConnell, Feldman, Aunos, and Prasad (2011) [37]	Parental cognitive impairment and child maltreatment in Canada.
S29—Sinanan (2011) [38]	The impact of child, family, and child protective services factors on reports of child sexual abuse recurrence.

**Table 2:** Family risk factors.

Category	Risk Factors	Study
Patterns of social and economic interaction	Hazardous housing	S1, S8, S13, S15
	Low social support	S4, S9, S11, S12, S13, S16, S20, S28
	No means of transportation	S8
	Mismanaged finances	S2, S4, S5, S7, S9, S10, S11, S12, S14, S15, S17, S20, S26, S27, S29
	Violence in the community and neighbourhood	S8, S9, S23
Family characteristics	History of allegations	S8, S9, S10, S14, S15
	Domestic violence	S3, S5, S6, S8, S9, S11, S13, S16, S17, S19, S21, S24
	Family disagreements and conflicts	S4, S7, S8, S11, S12, S15, S16, S17, S19
	Single-parent families	S3, S4, S7, S9, S10, S14, S17
	Multi-child family	S15, S17, S19, S24
	Multiple caregivers	S9
	Existence of stepmothers and stepfathers	S4, S9, S15
	Being the firstborn	S3
	Poor communication between separated parents	S8, S16, S26
Caregiver characteristics	Alcohol and/or drug abuse	S3, S5, S9, S10, S13, S14, S15, S17, S18, S22, S23, S26, S27
	Mental health problems	S5, S8, S9, S10, S11, S13, S14, S15, S17, S24, S26, S28
	History of inflicting maltreatment	S5, S26, S27
	Low level of education	S3, S9, S10, S12, S14, S15, S20
	Criminal record	S5, S9, S10, S11, S13, S14, S18, S25
	Lack of cooperation with CPS	S11, S12, S28
	Unintended pregnancy	S10, S14, S17
	History of having been maltreated	S3, S9, S10, S11, S13, S14, S21
	Age (under 18 years old)	S3, S6, S9, S10, S14, S15, S17, S19
Parenting	Unrealistic expectations towards the child	S11
	Negative attitudes towards the child	S10, S14, S16, S17
	Abusive and mistreating practices	S4, S5, S10, S11, S13, S14
	Multiple trips to children’s emergency room	S9, S11
	Parents absent in childrearing	S4, S8, S16, S21

## Data Availability

The data presented in this study are available on request from the first author.
